# Hydrogen Sulfide: A Therapeutic Option in Systemic Sclerosis

**DOI:** 10.3390/ijms19124121

**Published:** 2018-12-19

**Authors:** Amaal Eman Abdulle, Harry van Goor, Douwe J. Mulder

**Affiliations:** 1 Department of Internal Medicine, Division Vascular Medicine, University of Groningen, University Medical Centre Groningen, Hanzeplein 1, 9713 GZ Groningen, The Netherlands; d.j.mulder@umcg.nl; 2 Department of Pathology and Medical Biology, Section Pathology, University of Groningen, University Medical Centre Groningen, Hanzeplein 1, 9713 GZ Groningen, The Netherlands; h.van.goor@umcg.nl

**Keywords:** gasotransmitters, systemic sclerosis, vasculopathy, endothelial injury, therapeutic intervention

## Abstract

Systemic sclerosis (SSc) is a lethal disease that is characterized by auto-immunity, vascular injury, and progressive fibrosis of multiple organ systems. Despite the fact that the exact etiology of SSc remains unknown, oxidative stress has been associated with a large range of SSc-related complications. In addition to the well-known detrimental properties of reactive oxygen species (ROS), gasotransmitters (e.g., nitric oxide (NO), carbon monoxide (CO), and hydrogen sulfide (H_2_S)) are also thought to play an important role in SSc. Accordingly, the diverse physiologic actions of NO and CO and their role in SSc have been previously studied. Recently, multiple studies have also shown the importance of the third gasotransmitter H_2_S in both vascular physiology and pathophysiology. Interestingly, homocysteine (which is converted into H_2_S through the transsulfuration pathway) is often found to be elevated in SSc patients; suggesting defects in the transsulfuration pathway. Hydrogen sulfide, which is known to have several effects, including a strong antioxidant and vasodilator effect, could potentially play a prominent role in the initiation and progression of vasculopathy. A better understanding of the actions of gasotransmitters, like H_2_S, in the development of SSc-related vasculopathy, could help to create early interventions to attenuate the disease course. This paper will review the role of H_2_S in vascular (patho-)physiology and potential disturbances in SSc. Moreover, current data from experimental animal studies will be reviewed. Lastly, we will evaluate potential interventional strategies.

## 1. Introduction

Systemic sclerosis (SSc) is a severe progressive fibrotic connective tissue disease that often affects multiple systems [1,2]. The disease is characterized by chronic inflammation, autoimmunity, and vasculopathy, which all lead to substantial disability, high morbidity and mortality rates, and a decreased quality of life [3]. Reported prevalence rates for SSc range from 30 to 300 cases per 1 million persons, and the age of onset is approximately 50 years [4,5,6]. A variety of disabling complications (e.g., skin fibrosis, gastrointestinal dysmotility, scleroderma renal crisis (SRC), and lung involvement) occur in the early stages of the disease. Despite many efforts, the pathogenesis still remains unknown and effective disease-modifying treatment is currently unavailable. In addition, current treatment options (such as immunosuppressants) can cause a variety of adverse effects, including bone marrow depression, impaired hepatic and renal function, and infections. Therefore, new (low-risk) therapy options need to be found.

Endothelial dysfunction is a main event in the pathogenesis of SSc [7], and the vascular bed is a major target for immune-inflammatory injury. The vascular injury (i.e., vasculopathy) is characterized by thedysfunction of endothelial cells (ECs), inflammation, and vascular wall remodeling [8]. Raynaud’s phenomenon (RP), which manifests as episodic vasospastic events leading to the discoloration of the extremities, is the most recognizable and primary clinical sign that reflects the vascular injury [9]. Raynaud’s phenomenon often precedes visceral disease by several years and, therefore, RP is a crucial part of the important criteria for the diagnosis of early SSc (VEDOSS criteria) [2]. Following RP, the vasculopathy often progresses and becomes clinically evident as necrotic lesions of the fingertips (i.e., digital ulcerations), SRC, and pulmonary arterial hypertension (PAH) [2]. A current hypothesis is that early detection of SSc may allow an adequate start of appropriate therapeutic regimen to arrest disease progression.

The exact etiology of SSc remains unknown, but some studies have suggested that gasotransmitters play an important role in its pathogenesis. Gasotransmitters, which are endogenously produced gaseous signaling molecules, play a major role in several physiological processes, ranging from vessel tone regulation, modulation of the immune system, and protection against oxidative stress [10,11]. To date, three main gasotransmitters have been discovered, namely, hydrogen sulfide (H_2_S), carbon monoxide (CO), and nitric oxide (NO) [12]. As these gases perform critical roles, a deviation of their normal level may be associated with the occurrence of several diseases ranging from neurodegenerative diseases to auto-immune diseases. Interestingly, some reports have stated that SSc patients show a decreased level of H_2_S. Moreover, the beneficial effects of H_2_S in a scleroderma-like experimental animal study have also been proven. Therefore, an assessment of the role played by H_2_S in SSc-related vasculopathy could lead to a better understanding of this disease, which could subsequently help to create early interventions to attenuate the disease course. This review focuses on the role of H_2_S the pathogenesis of SSc-related vasculopathy in relation to its functional properties in the healthy vascular system. Furthermore, we evaluate the effects of H_2_S-based therapeutic interventions.

## 2. Role of Nitric Oxide and Carbon Monoxide in Systemic Sclerosis

The role of NO in SSc has been extensively discussed elsewhere [13,14,15,16,17,18], and, therefore, will be shortly mentioned here. The free radical gas NO is synthesized from the amino acid L-arginine by a family of isoenzymes, termed nitric oxide synthases (NOS) [19,20]. There are three known isoforms, namely endothelial NOS (eNOS), neuronal NOS (nNOS), and inducible NOS (iNOS). Endothelial NOS is the major isoform regulating vascular function, and it is primarily expressed in ECs, cardiomyocytes, neurons, and hepatocytes [21]. Induction of iNOS occurs usually in response to infection and chronic inflammation [22], and it is especially found in immunological cells, such as macrophages and neutrophils [23,24]. Nitric oxide may stimulate soluble guanylyl cyclase (sGC) in vascular smooth muscle cells (VSMCs) to induce the formation of cyclic guanosine monophosphate (cGMP) [25,26]. Subsequently, the formation of cGMP activates protein kinase G (PKG) [26], which leads to the reduction of cytosolic Ca^2+^ and reduction of the sensitivity of smooth muscle cells (SMCs) to Ca^2+^. As a result of this process, the intracellular concentration of calcium decreases and vasorelaxation is promoted [11,27,28]. Endothelial dysfunction is believed to be a main target in SSc. Although several mechanisms have been proposed that play an important role in this process, the exact role of NO still remains incompletely understood as both protective and harmful effect of NO have previously been described [13]. It was previously suggested that a decreased release of NO, which may lead to impaired endothelial-dependent vasodilatation, might play an important role in the pathogenesis of the SSc-related vasculopathy. In support, several studies reported a decreased level of NO release [29,30]. Subsequently, this may promote the development of vasculopathy by inducing recurrent RP attacks, increasing vascular wall thickness, and by enhancing the platelet aggregation. Moreover, NO is also believed to protect the endothelium from oxidative stress by acting as a free radical scavenger, and inhibiting lipid peroxidation [31,32]. In contrast, NO is also believed to have several harmful effects. For instance, it was previously reported that iNOS expression, which is often considered as a harmful enzyme [33], is up-regulated in affected skin, fibroblast, and endothelial cells [14,34]. Interestingly, it has been proposed that with the progression of SSc the NO production, which is primarily produced by eNOS in the early stages of the disease, is replaced by iNOS in endothelial cells and fibroblast [14,34]. This finding may indicate that the increased levels of NO, which were previously reported in SSc [35], may be induced by the increased expression of mainly iNOS. These conflicting results indicate that the exact role of NO in SSc still remains complex, and further studies should be conducted in order to clarify the role of NO in the pathogenesis of SSc.

Although the role of CO in SSc is less known, previous studies have suggested that CO may play an important role in several vascular diseases with a similar pathology as SSc. Carbon monoxide is generated by haem oxygenases (HO), namely HO-1 and HO-2, which are both expressed in the heart and blood vessels, as a result of the degradation of haem [36,37]. The oxidative degradation of haem, catalyzed by HO, results in the formation of iron (Fe^2+^), CO, and biliverdin [27]. Biliverdin is further converted into biologically important antioxidant bilirubin. Comparable to the formation of NO, this reaction requires O_2_ and nicotinamide adenine dinucleotide phosphate (NADPH) as cofactors. Similar to NO, CO is also thought to have important vasoregulatory effects. For instance, CO is thought to induce vasorelaxation in large vessels (e.g., aorta) by activating sGC in VSMCs, which leads to the formation of cGMP [27]. In smaller vessels, CO is thought to promote vasorelaxation by affecting calcium-activated potassium channels in VSMCs [38]. In contrast to these findings, other studies failed to demonstrate the vasodilatory effect of CO on cerebral arteries [39,40], or even reported on the vasoconstricting effect of CO [41], presumably by negatively affecting eNOS activity [28,42]. In addition to its vasoregulatory effect, CO is also believed to have several anti-inflammatory, anti-apoptotic, and pro-apoptotic effects. For instance, it was previously shown that CO may act as an anti-apoptotic agent in ECs, and, thus, prevent cell injury through AKt activation, and by affecting p38 mitogen-activated protein kinases (MAPK) signaling pathway [43,44,45]. In contrast, CO can also arrest fibroblast proliferation [46] as well as VSMC proliferation [47]. Considerable evidence supports a protective role for the HO-1/CO system in several cardiovascular diseases (e.g., atherosclerosis, myocardial infarction, and hypertension). The protective effect of CO in these diseases is thought to be associated with the prevention of oxidative stress-induced endothelial damage [43], increased production of bilirubin [48], increased stability of HIF-1α [49], and improved vasorelaxation [38]. Given the fact that some of these diseases may share a common pathogenesis, it could be postulated that CO might also have several beneficial effects in SSc patients. 

## 3. The Role of Hydrogen Sulfide in Vascular Biology

### 3.1. Production of H_2_S

Hydrogen sulfide is an evolutionary gaseous signaling molecule, and the earliest forms of life on earth depended on sulfur as an energy source and not oxygen. Only at the time that oxygen levels started to rise on earth, sulfide syntrophy largely disappeared. It was not until the last couple of decades that H_2_S was rediscovered in mammalians as being an endogenously produced gasotransmitter, and its important functions have now been increasingly recognized [50]. Although high concentrations of H_2_S are believed to be toxic, the enhancement of its physiological effects has been proven to be beneficial in several diseases.

**Figure 1:**
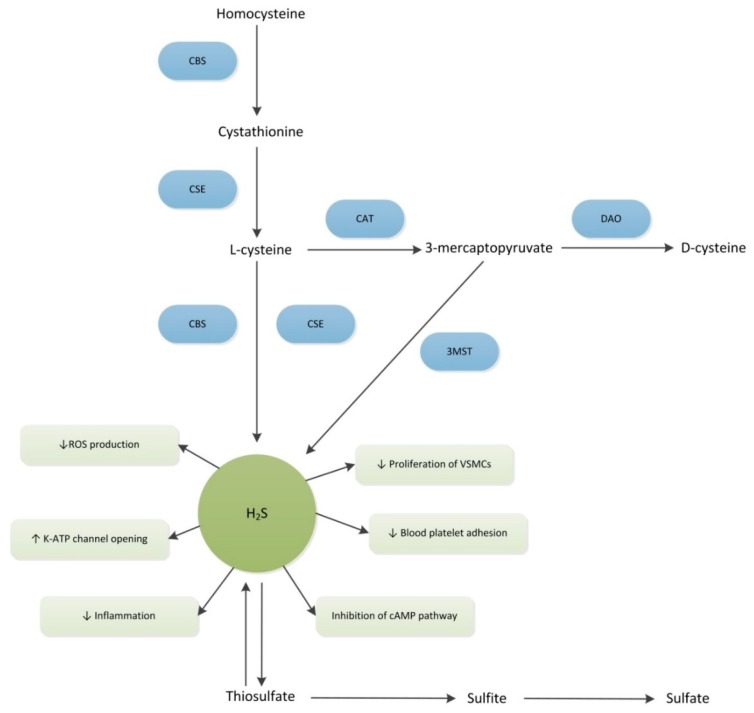
Schematic presentation of the production and function of hydrogen sulfide. Cystathionine β-synthase (CBS); cystathionine γ-lyase (CSE); cysteine aminotransferase (CAT); d-Amino acid oxidase (DAO); 3-mercaptopyruvate sulfurtransferase (3MST); reactive oxygen species (ROS); vascular smooth muscle cells (VSMCs); ATP-sensitive potassium channel (K-ATP); cyclic adenosine monophosphate (cAMP); Up-arrows represent up-regulations, down-arrows represent down-regulation.

This endogenous gasotransmitter is enzymatically generated by four enzymes, namely cystathionine β-synthase (CBS), cystathionine γ-lyase (CSE), cysteine aminotransferase (CAT), in conjunction with 3-mercaptopyruvate sulfurtransferase (3MST) [51,52,53,54]. These H_2_S producing enzymes are expressed in various vascular cell types (e.g., ECs and vascular smooth muscle cells (VSMCs)). Both CBS and CSE are found in the cytosol, whereas 3MST is mainly found in the mitochondria [55]. In ECs, CSE activity, which is strictly regulated by intracellular Ca^2+^ concentrations, is the main contributor to H_2_S production [56]. Both of the pyridoxal-5 phosphate-dependent enzymes (i.e., CSE and CBS) play an important role in the transsulfuration pathway [55,57]. During this process, homocysteine is converted into cystathionine by CBS, which is then converted by CSE to L-cysteine (Figure 1). Ammonium and pyruvate are also produced during this process. However, their exact roles in cellular functions remain not well clarified [58]. Although CSE, CBS, 3MST, and CAT are essential for the formation of H_2_S, it should be realized that these enzymes are also responsible for a number of other reactions that do not lead to the production of H_2_S [57]. Although H_2_S can also be produced through several non-enzymatic processes (e.g., through reductive chemistry of various forms of sulfur, including thiosulfate, thiocystine, and sulfite), these pathways are less well understood and only account for a small portion of H_2_S production [59]. 

The concentration of H_2_S in human blood was previously estimated to range from 10–100 μM [60,61], however, recent studies using more precise methods reported values of only 14–15 nanomolar [62,63]. Moreover, it should be noted that the measured H_2_S concentration is strongly dependent on the age of the subject and the method used [64]. 

Hydrogen sulfide can be quickly eliminated and it is initially oxidized to thiosulfate. Following this process, thiosulfate is further converted into sulfite and sulfate (Figure 1). Other routes of elimination include binding to methemoglobin and cytosolic methylation [65]. Given the fact that thiosulfate can easily be converted back into H_2_S, this sodium salt is often considered as a H_2_S donor. Sulfate is considered an important end-product of H_2_S metabolism. In humans, the majority of the circulatory inorganic sulfate is generated from the sulfur containing amino acids (SAA) methionine and cysteine, which are both derived from dietary protein [66,67]. However, because of the beneficial effects of H_2_S, it can also be hypothesized that the higher intake of SAAs contributes to the formation of H_2_S, and thereby, beneficially influences the cardiovascular profile, and, as a result, patient survival [68,69]. Therefore, sulfate levels could also possibly serve as an indication of H_2_S production. However, in order to understand the role of this important gasotransmitter in pathophysiology of many vascular diseases (including SSc), one has to comprehend the role that it plays in normal vascular biology. 

### 3.2. Vascular Biology

#### 3.2.1. Vascular Tone Regulation

Regulation of the vascular tone is crucial in maintaining adequate blood flow and tissue oxygenation to the organs. This balance in vasodilation and vasoconstriction is strictly regulated by various factors, among which H_2_S plays an important role. Hydrogen sulfide is believed to have a biphasic effect on the vascular tone, through the mediation of both vasoconstriction and vasodilation. Lower concentrations of H_2_S are believed to induce vasoconstriction. This postulation is supported by the fact that the reversal of the vasodilatory effect of acetylcholine and histamine, which are both NO-dependent vasodilators, was achieved after treatment with H_2_S in lower concentrations (<100 µM) [70]. In addition, even very low concentrations of 30 µM were found to induce a strong vasoconstrictive effect. This vasoconstrictive effect is believed to be induced through the inactivation of NO, down-regulation of eNOS, increase in reactive oxygen species (ROS) production, and through the decrease of intracellular cyclic adenosine monophosphate (cAMP) in VSMCs [54,71]. Moreover, it was previously demonstrated that the vasoconstrictive effect of H_2_S was endothelial dependent in phenylephrine-precontracted rat aorta [72]. This experimental study demonstrated that the contractile effect of H_2_S disappeared after the removal of the endothelium. Interestingly, the contractile activity of H_2_S was found to be more prominent in the absence of oxygen [73].

**Figure 2:**
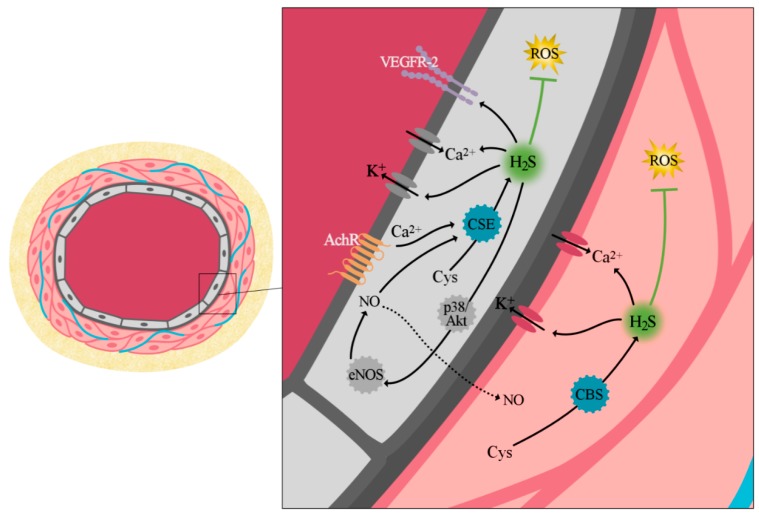
Physiological effects of intracellular hydrogen sulfide production in vascular smooth muscle cells and endothelial cells. Vascular endothelial growth factor receptor 2 (VEGFR-2); Acetylcholine receptor (AchR); Cysteine (Cys); endothelial nitric oxide synthases (eNOS); black solid arrows represent a direct effect, the black dotted arrows represent a direct effect on a different cell, and the green T-bar represents the inhibitory effect of hydrogen sulfide.

Conversely, higher levels are believed to promote vasodilation by affecting K_ATP_ channels, Ca^2+^ channels, and the Cl^−^/HCO_3_^−^ exchanger (Figure 2). In particular, H_2_S leads to the hyperpolarization of K_ATP_ channels in VSMCs, which reduces intracellular Ca^2+^ and leads to the relaxation of smooth muscle cells [74]. However, given the fact that glibenclamide, which is a K_ATP_ inhibitor, only partially prevented vasodilation, it has to be noted that the vasodilatory effect of H_2_S is not solely exerted through its effect on the K_ATP_ channels [75]. In addition, H_2_S also induces vasodilation by modulating K_v_7 channels in VSMCs [75], and by affecting K_Ca_ channels [76]. Moreover, the inhibition of cytochrome c oxidase by H_2_S may lead to a decrease in cellular ATP levels and activate K_ATP_ channels, which subsequently promotes vasodilation [77,78]. In contrast to the vasoconstrictive effect of H_2_S, vasodilation is thought to be promoted by the activation of eNOS and stimulation of NO-release [79]. In addition, it has been shown that the intermediates that form due to the NO-H_2_S crosstalk might also play an important role in this process. Hydrogen sulfide has been proven to reduce the blood pressure in various experimental models. More particularly, several experimental animal studies in mice have shown a reduction of the blood pressure after the administration of both systemic and local H_2_S donors (e.g., NaHS) [80,81,82]. More recently, it has been reported that H_2_S and NO, which are both key regulators in various physiological functions, are able to interact with each other and form intermediates with important physiological functions [83]. For instance, H_2_S was found to reduce oxidized NO forms, and thereby form several intermediate products (e.g., SSNO^−^, HSNO, HNO, and HSSH), which are highly redox-sensitive [83]. Therefore, it has become increasingly clear that the interactions between NO and H_2_S may have a significant effect on the regulation of the vascular tone. In support, Hosoki et al. (1997) demonstrated that H_2_S can induce a stronger vasorelaxant effect in the presence of an NO donor [84]. Another study reported that HNO might induce vasodilation by activating the cGMP-dependent pathway [85]. A recent review describes these interactions between gasotransmitters in the redox system as the “redox signaling interactome”, and these interactions may lead to targets for new therapeutic interventions [86].

#### 3.2.2. Cell Proliferation and Angiogenesis 

Hydrogen sulfide was previously reported to stimulate EC proliferation, which is an essential part of the initial angiogenic response. Two independent studies reported that H_2_S, in various concentration (ranging from 1–600 µM), increased the proliferation of human umbilical vein endothelial cells (HUVECs) and of transformed endothelial cells by 30–100% [87,88]. Moreover, H_2_S, at low concentrations (10–20 µmol/L), promotes EC migration and adhesion, which may further stimulate angiogenesis. Regarding the angiogenic properties of H_2_S, Cai and colleagues (2007) reported that intraperitoneal administration of an H_2_S donor (NaHS, 10–20 µmol per kg/day for seven days) increased neovascularization in a mouse model [87]. However, higher concentrations of this H_2_S donor in the same assay failed to demonstrate this angiogenic property. This difference might be due to the bell-shaped biological dose-response, with lower doses having a cytoprotective effect, whereas a higher dosage might have a more pronounced cytotoxic effect [89,90]. 

There are several intracellular signaling pathways that play an important role in the pro-angiogenic effect of H_2_S. For instance, vascular endothelial growth factor (VEGF) plays a significant role in the angiogenesis, both in physiology and pathophysiology [91]. Moreover, it has previously been reported that VEGF promotes H_2_S synthesis, presumably through calcium/calmodulin-dependent activation of CSE [92]. Other important signaling pathways include the phosphatidylinositol 3-kinase–Akt–survivin pathway, the extracellular signal-regulated kinase, and p38 pathways [93]. In addition, there is some evidence supporting the role of NO in this process [94], however, its exact implication in disease must be further investigated.

#### 3.2.3. Antioxidant Effect of H_2_S

Hydrogen sulfide, in low concentrations, is believed to have a strong antioxidant capacity, which is mainly due to its ROS inhibiting and scavenging property. However, studies on its antioxidant mechanism are limited. Evidence exists indicating that H_2_S up-regulates glutathione (GSH) and increases the expression of antioxidant enzymes, such as glutathione peroxidase and superoxide dismutase [95,96,97]. In addition, H_2_S was reported to increase intracellular thioredoxin (Trx-1). This multifunctional molecule not only has ROS scavenging properties, but also plays an important role in cell proliferation [98], apoptosis [99], and gene expression [100]. More recently, increasing evidence revealed that the transcription factor nuclear factor like 2 (Nrf2) may regulate the antioxidant capacity of H_2_S by promoting cellular antioxidant gene expression (e.g., glutamate cysteine ligase regulatory subunit, glutamate cysteine ligase catalytic subunit) [101]. This transcription factor can also increase the expression of glutathione reductase, which promotes the recycling of GSH and increases GSH/GSSG ratio, and thereby, reduces oxidative stress [102]. 

## 4. The Potential Role of H_2_S in the Development of Systemic Sclerosis-Related Vasculopathy

### 4.1. Link between H_2_S and Vascular Injury

The vascular endothelium is considered to be a major target for pathological processes in SSc [7]. Damage to the endothelium is characterized by EC apoptosis, vascular endothelial permeability, and impairment of cell–cell adhesion, which may lead to alterations in EC signal transduction [103]. Collectively, this may lead to endothelial dysfunction, subsequently causing an impaired relaxation of the vessels. Patients often present with RP as the earliest manifestation of this endothelial dysfunction [104]. 

Homocysteine (Hcy), which is converted into H_2_S through the transsulfuration pathway, is considered to be a potential mediator in the initiation of endothelial dysfunction in SSc. It was previously reported that increased levels of Hcy are associated with a higher risk of several cardiovascular diseases, including atherosclerosis, renal, and cerebrovascular diseases [105]. Interestingly, Hcy is also found to be elevated in SSc patients [106,107,108]. The exact reason for this elevation is unknown. Although the causal relationship between genetic abnormalities and the development of SSc has never been demonstrated, it could be hypothesized that the elevated levels of Hcy may be caused by a defect in the transsulfuration pathway. For instance, Hankey et al. (1999) stated that the most common enzyme defect associated with moderately raised homocysteine level is a point mutation in the coding region of the gene for methylenetetrahydrofolate reductase (MTHFR) [109]. Szamosi et al. (2009) aimed to investigate the prevalence of the MTHFR gene polymorphism in SSc patients [110]. Although they did not demonstrate any differences in Hcy level and MTHFR genotypes, as compared to healthy controls, they did observe significantly higher levels of Hcy in patients with macroangiopathy. Therefore, they concluded that Hcy levels might be associated with the occurrence/development of SSc-related vasculopathy. 

**Figure 3:**
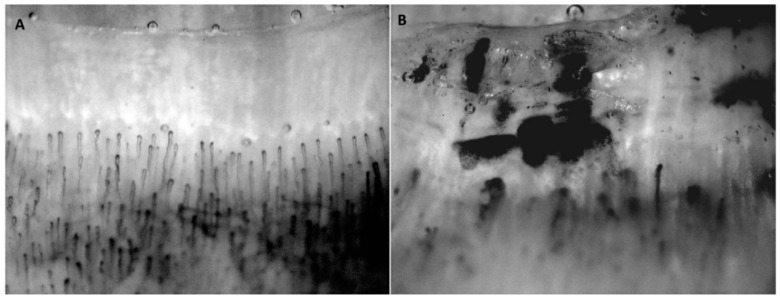
Nail-fold capillaroscopy patterns of a healthy subject (**A**) and a subject with systemic sclerosis (SSc) (**B**). The images of the nailfolds were taken at 180× magnification.

Moreover, elevated levels of Hcy can also be caused by a reduced vitamin intake or vitamin deficiency (e.g., folate, vitamin B12, vitamin B6) [111]. This defect could lead to a decreased production of H_2_S. A previously conducted experimental animal study in CBS deficient mice showed that *cbs*^+/−^ mice had a normal mean lifespan, and only developed a mild increase in plasma Hcy levels [112]. Moreover, other studies reported that *cbs*^+/−^ and *cbs*^−/−^ mice demonstrate endothelial dysfunction in both large and smaller vessels [113,114]. Hcy is believed to induce an oxidative milieu by stimulating the production of ROS (by the up-regulation of nicotinamide adenine dinucleotide phosphate) [115], and inhibiting antioxidant enzymes (including intracellular glutathione peroxidase) [116,117,118]. These superoxide anions may react with NO, which leads to the formation of peroxynitrite and a decreased bioavailability of NO, which may further impair the endothelial function [119]. The endothelial dysfunction in experimental animal studies can be largely attributed to the increase in oxidative stress and decrease of NO bioavailability. In addition, higher levels of Hcy may also lead to a decrease in H_2_S production, which is believed to be caused by a decrease in CSE activity. This decrease in CSE activity was previously demonstrated in an experimental animal study in which homocysteine at higher levels reduced CSE activity in rat liver [120]. Moreover, Chang et al. (2008) reported that, although cardiac CSE mRNA expression was up-regulated in the HHcy rats, CSE activity was inhibited by 50% [121]. Furthermore, the administration of H_2_S in this animal study slightly lowered Hcy level, decreased myocardial lipid peroxidation, and reduced ROS generation. The decrease in CSE activity may further increase the susceptibility to endothelial injury in SSc patients. The importance of Hcy in the development and perpetuation of endothelial injury is also supported by Caramaschi et al. (2007). They not only observed a higher level of Hcy in SSc patients, as compared to healthy controls, but also demonstrated that the plasma concentration of Hcy is strongly associated with nailfold videocapillaroscopy (NVC) patterns, which is commonly used to assess SSc specific microvascular lesions (Figure 3) [106,122]. This relationship was found to be irrespective of age, RP, SSc duration, the severity of skin involvement and folate levels. These findings are further supported by the observations of Motegi et al. (2014), stating that the plasma concentration of Hyc increases with the progression of nailfold abnormalities [123]. These observations may suggest that even mildly increased levels of Hcy may cause vascular damage in SSc through the direct effect of Hcy on the endothelium, and its inhibitory effect on H_2_S production. However, future studies are needed in order to clarify the exact role of Hcy in the occurrence of SSc-related vasculopathy.

It has to be noted that the levels of cysteine in healthy individuals are usually higher than the level of homocyteine. Therefore, it could be debated whether the contribution of homocysteine in the production of H_2_S is of great importance, in which slight deviations of the norm would lead to impaired H_2_S production. However, from a biochemical perspective, during hyperhomocysteinemia, homocysteine may compete with cysteine to bind to CSE, and therefore may indeed decrease H_2_S production [124]. Although there is no direct evidence indicating homocysteine induced reduction of CSE activity in SSc patients, a modified CSE activity, as proposed above, may indeed attenuate H_2_S generation.

The recurrent vasospastic events, known as RP, cause repetitive and prolonged ischemia followed by reperfusion (I/R injury), and may lead to substantial tissue hypoxia [125]. Some evidence exists indicating that hypoxia can up-regulate the expression of CSE, which may partly protect the cells from hypoxia-induced injury [126]. However, other reports have also shown decreased CSE expression as a result of suppressed expression of the transcription factor specificity protein 1 (Sp1) [127]. These conflicting observations underline the complexity of the role that is played by H_2_S in hypoxic conditions. Hydrogen sulfide is also believed to down-regulate hypoxia-inducible factor 1-alpha (HIF-1α) protein levels in cultured cells under hypoxic conditions [128]. This inhibitory effect was achieved with 100 μM of sodium hydrosulfide (NaHS) or 10 μM of sodium sulfide (Na_2_S).

Vascular injury in SSc may also be the result of impaired angiogenesis. Increasing evidence suggests that an imbalance of pro-angiogenic and anti-angiogenic mediators may be responsible for the impaired angiogenesis [129]. A previously conducted study demonstrated that ECs isolated from SSc patients show an impaired response to VEGF [130]. Hydrogen sulfide is believed to play a significant role in the essential components of the angiogenic response, including the proliferation and migration of endothelial cells. Two studies reported that H_2_S leads to an increase of 20% in the proliferation of transformed endothelial cells, and 100% increase in HUVEC cells, in addition to a 30% increase in mitigation in transformed endothelial cells and a six-fold increase in HUVEC cells [88,93]. The pathways that are involved in these processes have been studied, and H_2_S was found to activate PI-3K/Akt axis, enhance the phosphorylation of MAPK pathway, and induce hsp27 phosphorylation [87,88]. Furthermore, it is thought that K_ATP_-channel opening, more particularly K^+^ efflux, can promote EC motility [88]. In addition, it was previously demonstrated that VEGF increases the production of H_2_S, which may subsequently lead to VEGF-induced and H_2_S-induced EC migration. A current hypothesis is that both VEGF and H_2_S-induced EC migration involve the same downstream pathways, and this assumption is supported by Papapetropoulos et al. (2009), who reported that “VEGF-induced angiogenesis was suppressed in aortic rings of CSE-deficient mice” [88].

### 4.2. Link between H_2_S and Inflammation

Endogenously synthesized H_2_S is believed to be an important mediator of inflammation [131], and therefore may play an important role in the pathogenesis of SSc-related vasculopathy. Current literature has provided some conflicting results on the exact role of H_2_S in the inflammatory process. For instance, H_2_S is thought to have both pro-inflammatory and anti-inflammatory effects. This is presumably due to the biphasic effect of H_2_S, with low concentrations inhibiting inflammation and higher concentrations inducing inflammation. The pro-inflammatory effect is thought to be induced by the activation of adenosine triphosphate (ATP)-sensitive K^+^ channels, and this was previously observed in mice with septic shock [132]. The administration of a CSE inhibitor, which decreases the H_2_S level, was shown to increase survival in this experimental study [132]. Moreover, in an experimental animal study, Zanardo et al. (2006) demonstrated that H_2_S donors inhibit leukocyte adherence during vascular inflammation, which further supports the anti-inflammatory effects of H_2_S [133]. In another experimental animal study, the administration of H_2_S donors, such as NaSH, was found to induce inflammation through the up-regulation of nuclear factor-kappa B (NF-κB) and extracellular-signal-regulated kinase (ERK) 1/2 signaling [134]. Furthermore, Rinaldi et al. (2006) found that H_2_S prevented apoptosis of human granulocytes via the inhibition of p38 mitogen-activated protein kinases and caspase 3 [135]. Conversely, H_2_S is also thought to have several anti-inflammatory effects, including free radical scavenging properties (e.g., peroxynitrite), inhibition of leukocyte adherence to the endothelium [133], and H_2_S is believed to reduce the leukocyte infiltration [136,137].

### 4.3. Link between H_2_S and Fibrosis

Hyperhomocysteinemia might be a contributing factor in the increased production of collagen in SSc patients [138]. Hyperhomocysteinemia-induced oxidative stress is thought to be a key player in this process. During this process, matrix metallo-proteinases (MMPs) are believed to be activated, which subsequently cause disturbances in the metabolism of extracellular matrix (ECM). Moreover, Hcy is believed to lead to vascular stiffness by regulating the elastin/collagen ratio [139]. As increased levels of Hcy are known to decrease the CSE activity, it could be postulated that hyperhomocysteinemia leads to a decreased production of H_2_S, which may further sustain the pro-fibrotic state [121]. Conversely, hydrogen sulfide itself is believed to have several anti-fibrotic effects. For instance, H_2_S is thought to inhibit myofibroblast activation, which is a key event in the pathogenesis of fibrosis in SSc [140]. In addition, the anti-inflammatory effect of H_2_S (e.g., decreasing the expression of inflammatory cytokines) is also thought to block/reduce the disease-related pro-fibrotic state. Wang and colleagues demonstrated that NaHS, which is an H_2_S donor, inhibits the fibroblast differentiation and attenuates the protein expressions of TGF-β1 [141]. In support, several others have concluded “that organ fibrosis might be associated with H_2_S deficiency” [142,143]. The blockage of TGF-B1-induced fibroblast differentiation is presumably achieved through the inhibition of the phosphorylation of Smad3 and mitogen-activated protein kinases [144]. Furthermore, ROS are also thought to play a crucial role in the formation of fibrosis in SSc patients [8]. Hydrogen sulfide has been demonstrated to have ROS-scavenging properties, and thereby eliminates the fibrosis-inducing properties of ROS. Moreover, it was previously shown that the administration of an H_2_S donor can lead to the increased expression of antioxidant enzymes (e.g., manganese superoxide dismutase, catalase, and copper-zinc superoxide dismutase), which reduces the oxidative milieu [145]. These findings are further supporting the suggestion that H_2_S might play a pivotal role in the pathogenesis of SSc-related vasculopathy.

## 5. Hydrogen Sulfide as a Therapeutic Option

Exogenous alterations of H_2_S levels can be done in several ways, including, but not limited to, treatment with sulfide-sodium salts, exposure to gaseous H_2_S, slow-releasing H_2_S donors, and by improving the endogenously produced H_2_S (e.g., up-regulating the expression of H_2_S-producing enzymes) [146]. It was not until the last couple of decades that the perception of H_2_S shifted and its important functions were recognized. Following this shift in perception, multiple studies have demonstrated the cytoprotective effects of H_2_S [136,137]. In particular, some have demonstrated that H_2_S, in relatively low concentrations, can up-regulate endogenous antioxidant systems, increase the production of cAMP, and can up-regulate anti-inflammatory and cytoprotective genes [147]. In contrast to lower concentrations, a higher concentration of H_2_S is believed to be cytotoxic, which is likely due to its ability to inhibit cytochrome *c* oxidase. The inhibition of cytochrome c oxidase in complex V leads to a decrease in ATP production, and in the presence of oxygen it may lead to the formation of ROS [148]. Moreover, the generation of ROS, glutathione depletion, and the induction of mitochondrial cell death pathways may also enhance or even induce an oxidative milieu [149]. Although studies in SSc patients are lacking, several experimental animal and human studies in other vascular conditions have clearly demonstrated the beneficial effect of H_2_S treatment [74,150,151,152].

### 5.1. Hydrogen Sulfide in Experimental Animal Studies

Sulfide salts (e.g., Na_2_S and NaHS) have previously been used in numerous studies and demonstrated to have protective effects in a variety of diseases, including I/R-related diseases [153]. For instance, Zhang et al. (2013) demonstrated that preconditioning with NaHS reduced the I/R-induced hepatic injury in mice. They stated that the protective effect was presumably achieved by the inhibition of mitochondrial permeability transition pore (mPTP) opening [154]. Johansen et al. (2006) were the first to demonstrate the cytoprotective effect of H_2_S following myocardial I/R injury [155]. They reported that NaHS treatment (1 µM) 10 min prior to reperfusion led to a reduction of the infarct size of 20%. Following their results, several others reported supporting results. For instance, in a recently conducted study, researchers found that injection with Na_2_S (compared to vehicle) prior to the ischemia limited the extent of myocardial damage following I/R [156]. Moreover, they demonstrated that this sulfide salt reduced oxidative stress and apoptosis, increased the nuclear accumulation of Nrf2, increased the expression of Trx1 and HO-1, and activated the PKCε-p44/42-STAT-3 pro-survival signaling pathway [156]. Comparable results were found when NaHS was injected one day prior to the myocardial infarction (MI) [157]. NaHS not only reduced the myocardial infarct size, but also prevented apoptosis of cardiomyocyte. However, NaSH that was administered five days prior to MI had no effect. 

It was previously proposed that H_2_S might also have beneficial effects in renal IR injury. A previously conducted study demonstrated that topical treatment of NaHS on the kidneys 15 min prior to ischemia significantly increased the kidney function [158]. Another study reported that, during I/R, the renal injury is caused by a reduction in the CBS activity [159]. This finding may suggest that H_2_S may be hampered during I/R injury. Furthermore, subtoxic concentrations of H_2_S can induce a hibernation-like state in mice [160,161]. It was previously reported that mice, treated with gaseous hydrogen sulfide, showed a reduction of >90% in metabolic rate, and a decline in core body temperature to near ambient temperature. Moreover, H_2_S is also known to modulate the blood pressure, and, therefore, it may serve as an add-on treatment option in hypertension. In various experimental mice models, H_2_S was found to reduce the arterial pressure after systemic treatment with NaHS [162,163] or after local treatment [164]. These findings are supported by Huang et al. (2015), they stated that NaHS reduced systolic pressure in Dahl rats with high salt-induced hypertension [165].

It has become increasingly clear that H_2_S is also abundantly produced by gut microbiota through sulfate-reducing bacteria. Sulfate-reducing bacteria are a non-enzymatic source of H_2_S, and several bacteria (e.g., *E. coli*, *Salmonella*, and *Enterobacter*) are known to have the ability to produce H_2_S [166]. It was previously shown that antibiotic treatment aimed at reducing the intestinal microbiota resulted in a significant reduction of thiosulfate levels [167]. This finding sheds light on sulfate-reducing bacteria as a therapeutic target in various vascular diseases [166]. 

**Figure 4:**
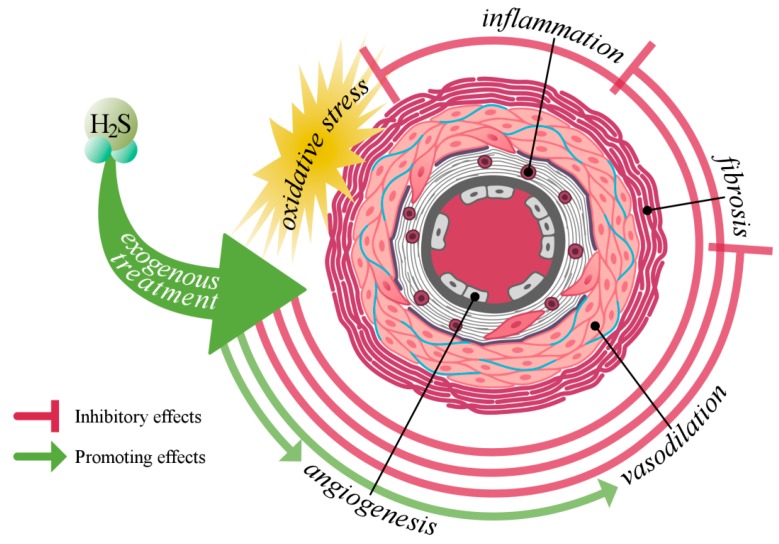
Hypothetical inhibitory and promoting effects of hydrogen sulfide treatment in the vasculature of SSc patients.

The only SSc-resembling experimental animal study was conducted by Wang and colleagues (2016), who investigated the effect of NaHS (56 and 112 μg/kg) in an experimental scleroderma-like animal study in mice [141]. They demonstrated that the plasma H_2_S levels were decreased in mice after injection with bleomycin. Moreover, they stated that H_2_S exerts an anti-inflammatory effect by the reduction of macrophage recruitment in lung tissues. In addition, they also stated that NaHS attenuates the protein expressions of TGF-β1, indicating that H_2_S also inhibits the fibroblast differentiation and extracellular matrix production. In support, several others have concluded “that organ fibrosis might be associated with H_2_S deficiency” [142,143]. These findings are further supporting the suggestion that H_2_S can be seen as a promising treatment option in SSc. The hypothetical effects of H_2_S treatment in SSc can be seen in Figure 4.

### 5.2. Hydrogen Sulfide in Human Studies

Despite the fact that several experimental animal studies clearly demonstrated the beneficial effects of treatment with sulfide salts, large studies in humans are still lacking. Thereby, the majority of H_2_S-releasing derivatives are not yet used in clinical settings. However, sodium thiosulfate (STS) is the exception, given the fact that this sodium salt is already used in various diseases. For instance, STS is a well-known effective antidote that is used in cyanide intoxication. Moreover, STS was proven to be beneficial as a treatment of cisplatin-induced hearing loss. For instance, an open-label, randomized trial demonstrated that hearing loss was significantly lower in children that were treated with STS, as compared to the control group [168]. Importantly, they also reported that the use of STS was not associated with the occurrence of serious adverse events. Sodium thiosulfate is also used in calciphylaxis in patients with end-stage renal disease [169]. Calciphylaxis is a rare disorder that is characterized by calcification of the tunica media, and it is often associated with high mortality rates. Calciphylaxis is a dreaded complication and it often leads to the ulceration of the extremities and contributes to the development of peripheral arterial disease [170]. This dreaded complication can also occur in SSc and it remains virtually untreatable. Several studies have shown that STS has beneficial effects on cutaneous lesions, with a potentially long-lasting effect (11 months–3 years) [171]. In a multi-centric study of 27 calciphylaxis patients that were treated with STS, 70% of these patients had a complete or partial response [172]. Although these effects are presumably mediated through different pathways as compared to the effects as hypothesized in SSc, these promising results indicate that STS can be safely used in the clinical setting. In our hospital, STS is already in a Phase III clinical trial that is designed to evaluate the effect of sodium thiosulfate compared with placebo treatment, in adjunction to optimal reperfusion therapy for acute MI, on left ventricular ejection fraction.

## 6. Conclusions

Vasculopathy is the hallmark of SSc and it often leads to severe disability and morbidity. To date, SSc is still associated with increased mortality rates given the fact that effective disease-modifying treatment options are currently unavailable. Hydrogen sulfide donors have previously been proven to have beneficial effects in a variety of diseases. These effects range from ROS scavenging, anti-fibrotic, pro-angiogenic, to vasoactive properties. Although the pathways that are involved in H_2_S production have not been clearly investigated in SSc, current data may suggest that the readily available H_2_S-donor STS may have a great therapeutic potential. Moreover, given the abundant experience in other diseases, with minimal adverse events reported, this drug could probably be safely administered in SSc. A current hypothesis is that early treatment of SSc with STS may alter or even arrest disease progression. Although future studies are needed in order to investigate this hypothesis, the promising effects of H_2_S-based therapeutic targets may encourage further study in SSc.
