# Correction: Abdulle, A.E., et al. Hydrogen Sulfide: A Therapeutic Option in Systemic Sclerosis. *Int. J. Mol. Sci.* 2018, *19*, 4121

**DOI:** 10.3390/ijms20225663

**Published:** 2019-11-12

**Authors:** Amaal Eman Abdulle, Harry van Goor, Douwe J. Mulder

**Affiliations:** 1 Department of Internal Medicine, Division Vascular Medicine, University of Groningen, University Medical Centre Groningen, Hanzeplein 1, 9713 GZ Groningen, The Netherlands; d.j.mulder@umcg.nl; 2 Department of Pathology and Medical Biology, Section Pathology, University of Groningen, University Medical Centre Groningen, Hanzeplein 1, 9713 GZ Groningen, The Netherlands; h.van.goor@umcg.nl

The authors wish to make the following correction to this paper [1].

Reference [8] refers to the wrong article. The correct reference [2] should be:

Abdulle, A.E.; Diercks, G.F.H.; Feelisch, M.; Mulder, D.J.; van Goor, H. The Role of Oxidative Stress in the Development of Systemic Sclerosis Related Vasculopathy. *Front. Physiol.* **2018**, *9*, 1–15, doi:10.3389/fphys.2018.01177.

The authors would like to apologize for any inconvenience caused to the readers by these changes.
